# Author Correction: Novel octapeptide containing the RGD sequence as a potential anti-SARS-CoV-2 agent: design, synthesis, and theoretical studies

**DOI:** 10.1007/s00726-026-03545-x

**Published:** 2026-07-04

**Authors:** Reinier Lemos, Orlando Ortiz, Luis Almagro, Kamil Makowski, Hortensia Rodríguez, Fernando Albericio, Margarita Suárez

**Affiliations:** 1 Laboratorio de Síntesis Orgánica, Facultad de Química, Universidad de la Habana, La Habana, 10400 Cuba; 2 Department of Surfactants and Nanobiotechnology, Institute for Advanced Chemistry of Catalonia. (IQAC-CSIC), Barcelona, 08034 Spain; 3 Centro de Investigación Biomédica en Red Bioingeniería Biomateriales y Nanomedicina (CIBER-BBN), Madrid, 28029 Spain; 4 School of Chemical Sciences and Engineering, Yachay Tech Medicinal Chemistry Research Group (MedChem-YT), Yachay Tech University, 100119 Urququi, Ecuador; 5 Peptide Science Laboratory, School of Chemistry and Physics, University of KwaZulu-Natal, Durban, 4001 South Africa; 6 Department of Organic Chemistry, University of Barcelona, Barcelona, Spain

**Author Correction to: Amino Acids (2025) 57:56**

10.1007/s00726-025-03480-3

In this article the author’s name Reinier Lemos was incorrectly written as Reiner Lemos. The original article has been corrected.

